# Nonfunctional alleles of long‐day suppressor genes independently regulate flowering time

**DOI:** 10.1111/jipb.12383

**Published:** 2015-09-17

**Authors:** Xiao‐Ming Zheng, Li Feng, Junrui Wang, Weihua Qiao, Lifang Zhang, Yunlian Cheng, Qingwen Yang

**Affiliations:** ^1^ National Key Facility for Crop Gene Resources and Genetic ImprovementInstitute of Crop Sciencesthe Chinese Academy of Agricultural SciencesBeijing100081China; ^2^ College of Life SciencesHubei UniversityWuhan430062China

**Keywords:** Domestication, flowering time, long‐day suppressor genes, loss‐function allele, rice

## Abstract

Due to the remarkable adaptability to various environments, rice varieties with diverse flowering times have been domesticated or improved from *Oryza rufipogon*. Detailed knowledge of the genetic factors controlling flowering time will facilitate understanding the adaptation mechanism in cultivated rice and enable breeders to design appropriate genotypes for distinct preferences. In this study, four genes (*Hd1*, *DTH8*, *Ghd7* and *OsPRR37*) in a rice long‐day suppression pathway were collected and sequenced in 154, 74, 69 and 62 varieties of cultivated rice (*Oryza sativa*) respectively. Under long‐day conditions, varieties with nonfunctional alleles flowered significantly earlier than those with functional alleles. However, the four genes have different genetic effects in the regulation of flowering time: *Hd1* and *OsPRR37* are major genes that generally regulate rice flowering time for all varieties, while *DTH8* and *Ghd7* only regulate regional rice varieties. Geographic analysis and network studies suggested that the nonfunctional alleles of these suppression loci with regional adaptability were derived recently and independently. Alleles with regional adaptability should be taken into consideration for genetic improvement. The rich genetic variations in these four genes, which adapt rice to different environments, provide the flexibility needed for breeding rice varieties with diverse flowering times.

**Edited by:** Jianqiang Wu, Kunming Institute of Botany, CAS, China

## INTRODUCTION

Yield improvement has always been an important goal of scientific research. Improvement in crop productivity requires optimal flowering time for wide geographical adaptation as well as for various management conditions (Izawa 2007; Wu et al. 2013). *Oryza rufipogon*, the common ancestor of cultivated rice, grows in the tropics; yet current rice varieties have been domesticated to grow widely from 53 °N to 40 °S (Khush 1997; Brambilla and Fornara 2014). Due to the remarkable adaptability to various environments, rice varieties with diverse flowering times have been domesticated or improved from their ancestry. For example, the two major rice subspecies, *indica* and *japonica,* were domesticated to adapt to different ecological and geographical environments (Chou 1948; Caicedo et al. 2007). Some elite rice cultivars flower extremely early with weak photoperiod sensitivity in order to adapt to short summer growing seasons (Fujino and Sekiguchi 2005; Wei et al. 2008; Li et al. 2013). Optimal flowering time is an important breeding objective, which enables rice to adapt to seasonal changes and make maximum use of temperature and sunlight resources (Izawa 2007). Detailed knowledge of the genetic factors that control rice flowering time will increase our understanding of the adaptive mechanisms in cultivated rice and enable breeders to design appropriate genotypes for distinct preferences (Putterill et al. 2004).

Recent advances in flowering time research in rice have identified a more complex and unique flowering pathway compared with that of *Arabidopsis*, the model long‐day plant. Conserved *Heading date 1* (*Hd1*) and unique *Early heading date 1* (*Ehd1*) are two major floral signal integrators that receive multiple signals from other genes to control the expression of the florigens, *Heading date 3a* (*Hd3a*) and *RICE FLOWERING LOCUS T* (*RFT1*) (Tsuji et al. 2011). *Hd1* suppresses flowering in long‐day (LD) conditions, but activates it in short‐day (SD) conditions (Yano et al. 2000). *Ehd1* encodes a B‐type response regulator that may not have an ortholog in the Arabidopsis genome (Doi et al. 2004). *Ehd1* is an *Hd1*‐independent flowering pathway that promotes flowering in both LD and SD conditions, and is a critical convergence point of regulation by multiple signaling pathways (Doi et al. 2004). In SD conditions, *Ehd1* is controlled by *OsGIGANTEA* (*OsGI)*, *Early heading date 2* (*Ehd2*), and *OsMAD51*. In LD conditions, rice flowering is regulated by both the LD‐activation pathway and the LD‐suppression pathway (Tsuji et al. 2011). *OsMAD50*, *Ehd2*, *Early heading date 3* (*Ehd3*) and *Early heading date 4* (*Ehd4*), which promote flowering by directly or indirectly upregulating *Ehd1* expression, constitute a LD‐activation pathway in rice (Tsuji et al. 2011; Gao et al. 2013). On the other hand, *Ehd1* expression is inhibited by a number of negative regulators, including *Ghd7* (for *Grain number, plant height, and heading date 7*), *DTH8* (for *days to heading on chromosome 8*) and *OsPRR37* (for *Oryza sativa Pseudo‐Response Regulator 37*). Together with *Hd1*, these flowering‐time suppressor genes may constitute a LD‐suppression pathway in rice (Tsuji et al. 2011; Koo et al. 2013).

Rice is a facultative short‐day plant with a critical day length response and flowers rapidly in SD conditions (Tsuji et al. 2011). However, the growing area of domesticated rice, whose ancestral species grow in the tropics (SD conditions), was extended to high latitudes (LD conditions) following domestication and improvement (Izawa 2007). Domesticated rice can now flower under non‐inductive LD conditions (Komiya et al. 2008). Interestingly, many studies have found that the flower suppressor genes under LD conditions in rice are important for rice to extend to high latitude (Komiya et al. 2008; Xue et al. 2008). For example, molecular and association analysis revealed that natural variation in *Hd1* alleles contributes to variation in flowering time and plays an important role in the regional adaptation of rice (Komiya et al. 2008; Wei et al. 2013). Allelic variation at the *Ghd7* locus increases grain yield by adapting to long growing seasons and plays a key role in the adaptability of cultivated rice on a global scale (Xue et al. 2008; Lu et al. 2012). Sequence analysis of *OsPRR37* variants demonstrated that the variation contributes to the northward expansion of rice cultivation (Koo et al. 2013). However, how the natural variation of these loci is associated with flowering time in rice accessions remains unknown. In addition, their evolutionary patterns and relative importance within different rice cultivation areas are still not clear. Exploration of these issues will facilitate an understanding of the comprehensive role of each gene in flowering time regulation and provide an efficient way to improve ecological adaptation.

In this study, four genes (*Hd1*, *DTH8*, *Ghd7* and *OsPRR37*) in the LD‐suppression pathway of rice were collected and sequenced in the germplasm of 154, 74, 69 and 62 varieties, respectively, of cultivated rice (*Oryza sativa*) to reveal: (i) the level and pattern of sequence variation of the four loci between two rice subspecies, (ii) the relationship between polymorphism in the four loci and the natural variation in flowering time, and (iii) the roles of the four loci in flowering‐time regulation and the regional adaptability of rice.

## RESULTS

### Genetic variation in rice

**Figure 1:**
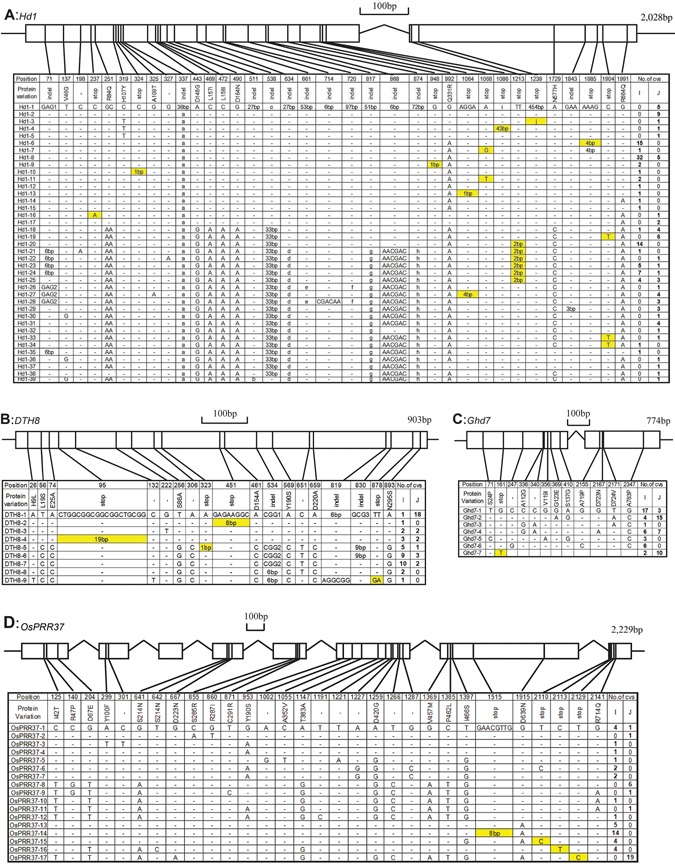
**Sequence polymorphism analysis of the *Hd1*, *DTH8*, *Ghd7* and *OsPRR37* coding regions in cultivars of rice** Wild types, which are the type of first accession in sequence alignment, are represented by dashes and variations that would lead genes to be nonfunctional are in yellow. The length of indels, which is more than 25, is represented by letter. The sequence information for each letter reads as follows. a:GCCAGGCGCCACCAGCGCGTCCCCGTCGCGCCGCTC; b:GACAACAGCAACAGCAACAGCAGCAAC; c:AACAACAACGACAACGACAATAACGACAACAAC; d:AGGTACAATTCGTACTACGACAACAAC; e:AATAACGACAACAGCAACAGCAACAGCAGCAACAACGACAACGACAACGACAA; f:TAACAACAACAGCAACAGCAGCAACAACGGCATGTATTTTGGTGAAGTCGATGAGTACTTTGATCTTGTCAGGTACAATTCGTACTACGACAACAAC; g:AATAACGACAACAGCAACAGCAACAGCAGCAACAACGACAACGACAACGAC; h:AATAACAACAACAGCAACAGCAGCAACAACGGCATGTATTTTGGTGAAGTCGATGAGTACTTTGATCTTGTC; i:ACCTTCACAGATCACAATGCTGAGTGAGCAGCAGCATAGTGGT; j:GTTAGGAAATAAACCTGACCGTGTGTGTTCAGGTCAGTTCGCGTGCGTCGTGTGTGTGTTTGTTGTTGAACGCGTCCGTCGTGTGGAGGTTCGTCCGAGTTTTAGCGCAGGCTAGCTAGCTGCGAGCGTGTGTATCGGGTTCAGTTGGAGAGTGTGGCGTCGTGCGCGGACGCTGGCGGCCGCGTCGTGCGCTGAGTTTGTGCTCATGCATGGCGTGAGCCGGGACGTGCGGATCGTGCGAGCAAAGGCCGGGGAAGGAGTTCGTGCTCCATTTGTGCGTCACCCTGGGCCTATATAAGCACATGTTCATCCCGTTGTTGATCACCGAGTGAAAAAAAAGAAAAGCCGGCATTCGTTGCCAGGGCGTTCGTTGCCATTGTCAAGCTTCACAAACTCTGCTACTCACTCTCGTGTTTGTGTGTGTCTCCGATCTTGGCCATTTCCAACATATAGT.

We sequenced and collected 359 sequences of four flowering suppressor loci under LD conditions from the representative germplasm (154, 74, 69 and 62 varieties for *Hd1*, *DTH8*, *Ghd7* and *OsPPR37*, respectively) of the two cultivated subspecies. The total lengths of the aligned coding regions for *Hd1*, *DTH8*, *Ghd7* and *OsPPR37* were 2,028, 903, 774 and 2,229 bp (Figure 1), respectively. The schematic diagrams and polymorphic sites of all four loci are shown in Figure 1. For the four nuclear loci, the species‐wide levels of variation in polymorphic sites varied from 12 (*Ghd7*) to 35 (*Hd1*) and the number of alleles ranged from 7 (*Ghd7*) to 39 (*Hd1*) in cultivated rice.

The entire *Hd1* coding region (2028 bp) was sequenced in the 154 varieties. Seventeen SNPs and 19 indels (inserts and deletions) were detected in the coding region of *Hd1*, of which 14 SNPs (82.35%) and 15 indels (78.95%) were located in the first exon. Nine indels led to changes in the open reading frame. Thirty‐nine alleles, named Hd1‐1 to Hd1‐39, were constructed (Figure 1A). The most prevalent alleles were Hd1‐2, Hd1‐6, Hd1‐8, Hd1‐19, Hd1‐20, Hd1‐23, Hd1‐24 and Hd1‐25, which included 9, 15, 37, 6, 14, 6, 8 and 7 varieties, respectively. Of the remaining alleles, each had five or less than five varieties. Five alleles (12.82%), Hd1‐8, Hd1‐18, Hd1‐23, Hd1‐24 and Hd1‐25, were shared by both cultivated subspecies.

For *DTH8* coding region (903 bp), 14 SNPs and six indels were detected in the 62 varieties (Figure 1B). *DTH8* with simple structure had only one exon and no intron. Of these polymorphic sites, three indels and three substitutions resulted in changes to four amino acids. Nine alleles designated as DTH8‐1 to DTH8‐9 were constructed based on these polymorphic sites. DTH8‐1, DTH8‐5, DTH8‐6 and DTH8‐7 were the most prevalent alleles, present in 19, 6, 12 and 12 varieties, respectively. These alleles were largely represented by most of the accessions of cultivated rice (80.64%). Six alleles (66.67%) were shared by both cultivated subspecies, except for DTH8‐2, DTH8‐8 and DTH8‐9.

The whole coding region of *Ghd7* was re‐sequenced in the 74 varieties. Twelve SNPs and no indel were detected in the 774 bp alignment (Figure 1C). Of these polymorphic sites, a 1 bp substitution in the first exon generated a loss functional allele. Seven alleles were constructed based on the SNPs. The alleles of Ghd7‐2 and Ghd7‐7 were largely represented by *japonica* varieties (80.65%) and Ghd7‐1, Ghd7‐4, and Ghd7‐6 were mainly represented by *indica* varieties (74.36%). The most prevalent alleles were Ghd7‐1, Ghd7‐2, Ghd7‐4 and Ghd7‐7, which were represented by 20, 19, 13 and 12 varieties, respectively. Of these, Ghd7‐7 was a nonfunctional allele largely represented by *japonica* varieties (83.33%).

Twenty‐nine SNPs and only one indel were detected in the 2229 bp coding region of *OsPPR37* (Figure 1D). Of these polymorphic sites, one indel and three SNPs resulted in amino acid changes. Seventeen *OsPPR37* types were classified according to these variations, designated as OsPPR37‐1 to OsPPR37‐17. The most prevalent alleles were OsPPR37‐8, OsPPR37‐14 and OsPPR37‐17, which were represented by 6, 14 and 19 varieties, respectively; while other alleles were rare types represented by only one to five varieties. Fourteen *indica* varieties (35%) possessed the OsPPR37‐14 allele with an 8 bp deletion, while 19 *japonica* varieties (65.52%) carried the OsPPR37‐17 allele.

Since the number of polymorphic sites is heavily dependent on sample and sequence size, we further computed the nucleotide diversity to compare genetic diversity in the four loci. Standard statistics of sequence polymorphism for four loci are shown in Table S1. The subspecies‐wide levels of silent nucleotide variation of exons varied across loci from 0.00239 (*OsPRR37*) to 0.00522 (*Hd1*) in *indica* and from 0.00201 (*OsPRR37*) to 0.00485 (*Hd1*) in *japonica*. Average silent nucleotide variation of *Hd1* was significantly higher than other loci (*P* < 0.05, for both *π* and *θ*).

### Contribution of function or loss function of the four loci to flowering‐time variation

**Figure 2:**
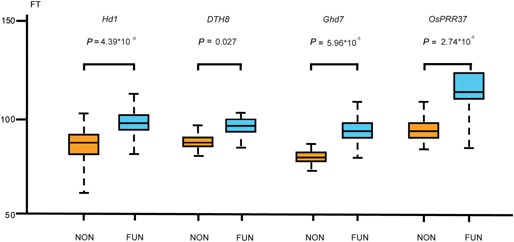
**Variation in flowering time of cultivars** Comparison of flowering times between accessions with nonfunctional and functional alleles. The yellow and blue bars represent nonfunctional (NON) and functional (FUN) alleles of *Hd1*, *DTH8*, *Ghd7* and *OsPRR37*, respectively. FT is the abbreviation of flowering time. *P* values were produced by two‐tailed *t*‐tests and Wilcoxon signed‐rank tests. Flowering time in each accession is mean ± SD (standard deviations).

Alleles with a premature stop codon or leading to changes in open reading frame were considered as potentially nonfunctional. We performed an association analysis to further understand the relationship between function or loss of function of the four loci and flowering‐time variation. However, population genetic structure can result in false associations between genetic markers and phenotypes. The already well demonstrated genetic differentiation between these two subspecies (Second 1982; Garris et al. 2005; Zhu et al. 2007) should be a source of a main strong genetic structure within the collection of varieties. So we divided the cultivated rice into two groups, including *indica* and *japonica*, for eliminating the effect of population structure on association analysis. Significant correlations in both *indica* (*Hd1*: *r *= 0.48, *P* = 1.98 × 10^−5^; *DTH8*: *r* = 0.31, *P *= 0.02; *Ghd7*: *r* = 0.64, *P *= 5.97 × 10^−5^; *OsPRR37*: *r *= 0.58, *P *= 2.85 × 10^−6^) and *japonica* (*Hd1*: *r *= 0.58, *P *= 2.98 × 10^−5^; *DTH8*: *r* = 0.41, *P* = 0.001; *Ghd7*: *r *= 0.44, *P *= 1.97 × 10^−5^; *OsPRR37*: *r* = 0.48, *P *= 1.85 × 10^−6^) were found between function or loss of function of the four loci and the variation in flowering time. At the same time, flowering time in varieties carrying nonfunctional type alleles was significantly shorter than that of varieties with functional type alleles (Figure 2 and Table S2). Significant differences in flowering time were examined among rice varieties with functional or nonfunctional alleles (*P *= 4.39 × 10^−6^ for *Hd1*; *P *= 0.027 for *DTH8*; *P *= 5.96 × 10^−5^ for *Ghd7*; *P *= 2.74 × 10^−5^ for *OsPRR37*; Figure 2).

For *Hd1* locus, a diverse collection of 154 *O. sativa* accessions was surveyed; nonfunctional alleles were detected in 74 (48%) of these accessions. Eight alleles were represented by more than five samples. Of these, six were nonfunctional alleles, and those varieties with all nonfunctional alleles had a shorter growing period (Figure 2). Average flowering time (FT) of accessions with nonfunctional alleles was about 76 d, which was significantly shorter (*P *= 4.39 × 10^−8^) than the other cultivated rice groups (99 d). Of the total FT diversity, about 28% was attributable to functional differences in the *Hd1* locus.

Four nonfunctional *DTH8* alleles were found in 13 accessions, accounting for 19% of the total accessions. More than five accessions had both DTH8‐4 and DTH8‐5. Flowering time in varieties with DTH8‐4 alleles had a significantly shorter growing period (FT = 63; *P *= 0.0001), but no difference in flowering time was observed between varieties with DTH8‐5 and other alleles. However, for functional and nonfunctional groups, average FT differed significantly (*P *= 0.027) at 76 and 99 d, respectively. Furthermore, about 16% of the total FT difference was attributable to divergence between functional and nonfunctional groups.

*Ghd7* is a major QTL for flowering time under LD conditions (Xue et al. 2008). Of the 74 varieties, 12 (16.21%) had a nonfunctional Ghd7‐7 allele and all accessions with Ghd7‐7 allele grew in temperate areas. Average FT in these samples was 58 d, approximately two‐thirds (60.42%) of the other varieties. 37% of the total FT variation was attributable to function or loss of function of the alleles.

*OsPPR37* was nonfunctional in 41 cultivated accessions, which accounted for 59% of the total population. OsPPR37‐14, OsPPR37‐15, OsPPR37‐16 and OsPPR37‐17 were nonfunctional alleles in the population. Average FT in varieties with these alleles was 78, 96, 97, and 97 d. Average FT in accessions with nonfunctional alleles was about 86 d which significantly differed (*P *= 2.74 × 10^−5^) from varieties containing functional alleles (104 d). 34% of the total FT variation was attributed to function or loss function of the alleles.

### Distribution and origin of the nonfunctional alleles

Nonfunctional alleles of the four loci were observed in both *indica* and *japonica* (Figure 1). Fifty‐two *indica* and 21 *japonica* varieties had *Hd1* nonfunctional alleles; nine *indica* and three *japonica* varieties had *DTH8* nonfunctional alleles; two *indica* varieties and 10 *japonica* varieties belonged to one cultivated rice type with Gdh7‐7, the only *Ghd7* nonfunctional allele; for the *OsPRR37* locus, nonfunctional alleles were detected in 19 *indica* and 22 *japonica* varieties. These results suggest that loss of function of suppressor genes plays an important role in the regulation of flowering time under LD conditions for the two subspecies of cultivated rice at the same time.

**Figure 3:**
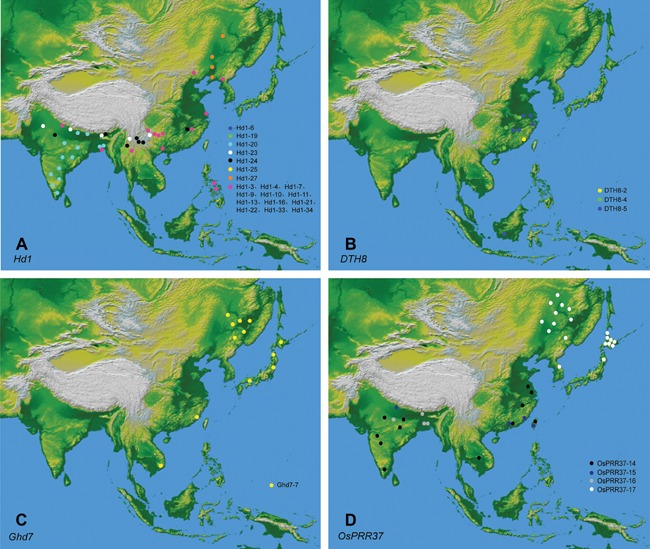
**Distribution map of rice landraces carrying the nonfunctional alleles** (**A**) *Hd1*. (**B**) *DTH8*. (**C**) *Ghd7*. (**D**) *OsPRR37*. Each circle represents one accession. Each color represents different haplotypes except that alleles with less than three varieties are shown in pink.

However, nonfunctional alleles of the four loci exhibited different geographical distribution patterns, and the nonfunctional alleles of each locus have distinct geographic origins (Figure 3). Varieties with the nonfunctional alleles *Ghd7* and *DTH8* are locally distributed in rice‐growing areas. Most of the varieties with Ghd7‐7 grow in northern China, the Korean Peninsula and Japan, and a number of *DTH8* nonfunctional alleles were found in varieties grown in east China. Varieties with *Hd1* and *OsPRR37* nonfunctional alleles were distributed throughout the rice‐growing areas (Figure 3). Yet, cultivars with different nonfunctional *Hd1* or *OsPRR37* alleles were mapped to regional regions of the rice‐growing areas. However, mantel's test detected a significant association between geographical distance and genetic distance (Fst/(1‐Fst)) in varieties with nonfunctional alleles for *Hd1* (*r* = 0.62, *P* = 0.019) and *OsPPR37* (*r* = 0.83, *P* = 1.23 × 10^−4^), but not in varieties with functional alleles for four loci (Figure S2; *Hd1*: *r* = 0.12, *P* = 0.17; *DTH8*: *r* = 0.21, *P* = 0.09; *Ghd7*: *r* = 0.24, *P* = 0.08; *OsPPR37*: *r* = 0.03, *P* = 0.51), suggesting that the isolation by distance model only applied to populations with the nonfunctional alleles *Hd1* and *OsPPR37*.

**Figure 4:**
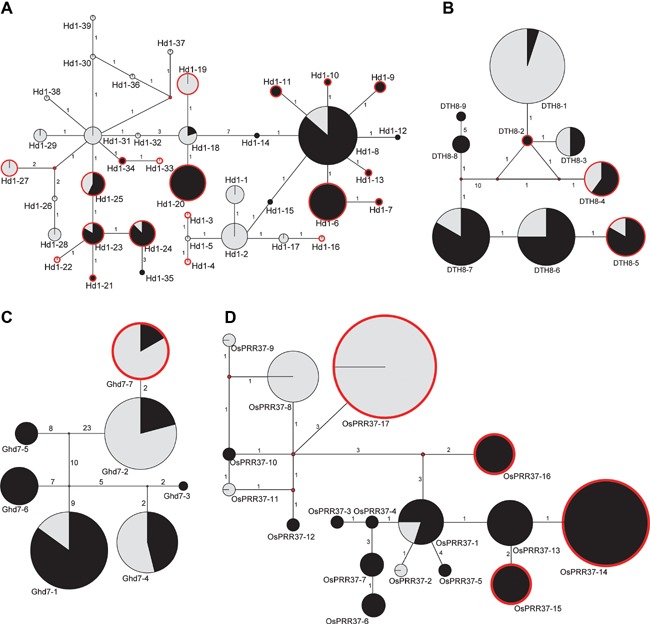
**Allele network analysis for four loci** (**A**) *Hd1*. (**B**) *DTH8*. (**C**) *Ghd7*. (**D**) *OsPRR37*. Allele frequencies are proportional to circle size. The proportions of the two rice subspecies (*indica* and *japonica*) are represented by gray and black, respectively. Circles with red borders indicate nonfunctional alleles and circles with black borders indicate functional alleles. Lines connect two alleles with the most recent relationships. The number on the lines between the circles indicates the evolutionary distance between the two alleles.

Network trees representing phylogenetic relationships in the four loci are shown in Figure 4. Except for *OsPRR37*, the most striking feature of the networks for the four loci (Figure 4) is that alleles with high frequency are functional types, with most old enough to have developed a star‐like branching structure (phylogenetic cluster). We found significant differences among the four loci in phylogenetic incompatibility (*Hd1*: *P* = 0.01; *DTH8*: *P* = 0.04; *Ghd7*: *P* = 0.02; *OsPRR37*: *P* = 0.01) using the JE method (Jakobsen and Easteal 1996). The results identified that three of the most frequent functional alleles corresponded to three major lineages of *Hd1*, and the other three genes possessed two major lineages. All of these functional alleles were located at the central position (Figure 4). The intermediate relationship of the functional alleles of the four loci suggests that these functional alleles might be ancestral (Figure 4). In addition, the nonfunctional alleles were distributed in different lineages. Twenty‐three of the 27 nonfunctional alleles of the four loci were located at the tips (Figure 4), which indicates that the nonfunctional alleles may have originated independently and recently (Bandelt et al. 1999).

## DISCUSSION

### Nonfunctional alleles of LD‐suppressor genes diversified flowering time in rice

We compared the coding region sequences of four suppressor genes for flowering time from 64–154 rice landraces and found many function‐loss mutations in all four genes. Significant correlations were detected between function or loss of function of the four loci and variation in flowering time. In addition, varieties with nonfunctional alleles flowered significantly earlier than those with functional alleles under LD conditions (Figure 2). These results indicate that function‐loss mutations in rice play an important role in the regulation of flowering time under LD conditions. Flowering time is one of the best‐studied ecologically significant traits under natural or artificial selection for adaptation of plants to specific natural environments. Some studies have found that loss of gene function is an important factor that regulates flowering time in many species. Of the flowering genes identified in *Arabidopsis*, *FRIGIDA* and *FLOWERING LOCUS C* are two suppressor genes of the vernalization pathway, and the loss of function of these two genes appears to underlie the extensive natural variation of flowering time for local adaptation (Le Corre et al. 2002; Balasubramanian et al. 2006). In wheat, one allele with a TE element insertion had the lowest expression level among the *Ppd‐D1* alleles, and this allele may be responsible for the longest growth period and for the adaptation to northern latitudes and higher altitude regions (Gao et al. 2009). In addition, large deletions in the *VRN‐1* first intron are associated with the spring growth habit in barley and wheat (Fu et al. 2005). Loss of gene function may change the regulatory network with minimal side‐effects, which may be an important reason why many loss‐of‐function mutations were detected in the regulatory network of flowering time (Hottes et al. 2013).

*Hd1*, *DTH8*, *Ghd7* and *OsPRR37* have different genetic effects on flowering time. *Ghd7* is the most important gene for adaptation to the northern region and the function or loss function of *Ghd7* explains 37% of the variation of flowering time in rice. *OsPRR37* and *Hd1*, which, respectively, explain 34 and 28% of the variation in flowering time in rice, are widely used in rice domestication and breeding; while cultivated rice varieties from different geographical regions contain different nonfunctional alleles in both loci. The function or loss function of *DTH8* explains 19% of the variation in flowering time and only fine‐tunes flowering time in rice. Alleles associated with regional adaptability should be taken into consideration for genetic improvement. Elucidation of the evolution patterns of these nonfunctional alleles as well as the relationship between them and the geographical distribution of rice provides opportunities to tailor rice environmental adaptability to suit diverse agricultural demands.

### Nonfunctional alleles of LD‐suppressor genes originated independently and recently

Based on multiple lines of evidence, we argue that the nonfunctional alleles of all four suppressor genes, which independently regulate flowering times in local rice varieties, originated from different ancestors during rice domestication or breeding under LD environments. Firstly, a clear regional distribution pattern in these nonfunctional alleles was observed. The distribution of the nonfunctional alleles *DTH8* and *Ghd7* affected the distribution of local rice varieties, while that of the other two genes (*Hd1* and *OsPRR37*) was closely correlated with latitude (Figure 3). Secondly, network studies suggested that the nonfunctional alleles were derived independently from distinct ancestral alleles with distant relationships (Figure 4). Thirdly, most nonfunctional alleles in different lineages were located at the tips (Figure 4); in addition, many phylogeographic studies have found that ancient alleles should be located at the center of the network tree, whereas recent alleles that are locally distributed geographically should be at the tips of the network tree. Taken together, our geographic analysis and network studies suggest that the nonfunctional alleles of these suppressor genes with regional adaptability were derived recently and independently.

The rich genetic variation in these four genes, which can adapt to different environments, provides the flexibility needed for designing various flowering times in rice. However, the mechanisms by which these genes regulate flowering time and their origin patterns in rice germplasm are still not thoroughly understood. Selection of genes in the photoperiod pathway from wild or landrace rice may enable cultivated rice varieties to adapt to different photoperiods along latitudes and farming systems (Ross Ibarra et al. 2007). Artificial selection would leave evolutionary footprints in the genomes of cultivated rice (Sabeti et al. 2006; Ross Ibarra et al. 2007). With wild rice samples included, these genes in cultivated rice will be analyzed in future studies and their evolutionary patterns compared with that of wild rice. This will shed new light on the domestication mechanism in rice, including the origin and formation of domestication traits and the specific pressures that affect these domestication traits, which can then be applied to design varieties of the desired quality.

## MATERIALS AND METHODS

### Plant materials

The sequences of *Hd1*, *DTH8*, *Ghd7* and *OsPRR37* (Table S3) were collected from five studies reported by Wei et al. (2013), Fujino et al. (2010), Wei et al. (2008), Lu et al. (2012) and Koo et al. (2013). To ensure the samples covering all distribution areas and rice subgroups, we further sequenced 5, 8, 7 and 14 accessions for *Hd1*, *DTH8*, *Ghd7* and *OsPRR37,* respectively. The varieties for phenotype identification of *Ghd7* and *DTH8* were grown in Wuhan and Nanjing in China, respectively, while the varieties for *OsPRR37* were grown in Korea. For the varieties detecting *Hd1*, we collected the phenotypes from the Chinese Crop Germplasm Information System in Beijing, China. All phenotypes of 359 accessions were obtained under LD conditions.

### DNA extraction, PCR amplification and sequencing

Fresh leaves were collected from field‐grown plants and genomic DNA was extracted using the hexadecyl‐trimethyl ammonium bromide method as described by Ge et al. (1999). We only amplified the coding region for each locus. Sequences were amplified from genomic DNA using LA Taq (Takara). Table S4 and Figure S1 provide a list of all primers used for polymerase chain reactions (PCRs) and sequencing. PCR amplification methods follow those in Zheng and Ge (2010). All accessions were sequenced by ABI3730XL automatic sequencer (Applied Biosystems). Since heterozygous individuals may exist for a small number of cultivars (Wei et al. 2013), PCR fragments of heterozygous samples were cloned into EASY vectors (Transgen, China) and three cloned DNA fragments were sequenced for each individual. Taq errors occur randomly so polymorphisms shared by more than two clones are unlikely to be artificial (Eyre et al. 1998; Zheng and Ge 2010). To further verify the singleton site, we re‐sequenced individuals that contained singletons in the alignments and obtained four clones after the second round of PCR.

### Statistical analysis

In this study, we classified haplotypes as functional or nonfunctional based on previous studies (Wei et al. 2008; Fujino et al. 2010; Lu et al. 2012; Koo et al. 2013; Wei et al. 2013). The correlation between the functional and nonfunctional of these alleles and flowering time was examined by the Spearman Correlation Coefficient Test. The differences of flowering time among varieties with different alleles were examined by ANOVA for each locus and, if significant (*P* < 0.05), the Duncan multiple range test and critical test conducted. A hierarchical analysis on flowering time differences was performed for the four loci by one‐way ANOVA, which estimated the relative contribution to flowering time variance of the function or loss of function of the suppressor genes. Statistical analysis was performed by R2.5.1.

### Phylogeographic analysis

Initial sequence data were assembled with the Contig‐Express and aligned using ClustalX 1.81 (Thompson and Toga 1997). Sequences were manually edited with DAMBE (Xia and Xie 2001). Insertions/deletions (indels) were included in the analysis. An allele network tree was constructed using the Median‐Joining model using network version 4.0 (Bandelt et al. 1999). Gaps with a length greater than one were considered a single mutation. The Mantel test performed in Arlequin version 3.5 was used to examine the correlation between geographical and genetic distance (Slatkin 1993).

## AUTHOR CONTRIBUTIONS

X.M.Z. and L.F. performed most of the research and X.M.Z. drafted the manuscript. J.W. carried out grafting experiments. W.Q., L.Z. and Y.C. carried out field experiments. Q.Y. designed the experiment, supervised the study, and revised the manuscript.

## Supporting information

Additional supporting information may be found in the online version of this article at the publisher's web‐site

**Figure S1**. Schematic diagrams of four nuclear loci and locations of the regions sequenced
Exons are shown as black boxes; thin lines between black boxes refer to introns. Locations of primers for each fragment are sketched above the diagrams using red arrows.

**Figure S2**. Distribution map of rice landraces carrying the functional alleles
(**A**) Hd1, (**B**) DTH8, (**C**) Ghd7 and (**D**) OsPRR37. Each circle represents one accession. Each color represents different haplotypes except that alleles with less than three varieties are shown in pink.

**Table S1**. Summary of nucleotide polymorphism of four loci

**Table S2**. The differences between the functional and nonfunctional alleles by one‐way ANOVA

**Table S3**. Sample list

**Table S4**. The primer sequences used in this study

## References

[jipb12383-bib-0001] Balasubramanian S , Sureshkumar S , Lempe J , Weigel D ( 2006) Potent induction of *Arabidopsis thaliana* flowering by elevated growth temperature. PLoS Genet 2: e106 16839183 10.1371/journal.pgen.0020106 PMC1487179

[jipb12383-bib-0002] Bandelt HJ , Forster P , Röhl A ( 1999) Median‐joining networks for inferring intraspecific phylogenies. Mol Biol Evol 16: 37–48 10331250 10.1093/oxfordjournals.molbev.a026036

[jipb12383-bib-0003] Brambilla V , Fornara F ( 2013) Molecular control of flowering in response to day length in rice. J Integr Plant Biol 55: 410–418 23331542 10.1111/jipb.12033

[jipb12383-bib-0004] Caicedo AL , Schaal BA ( 2004) Population structure and phylogeography of *Solanum pimpinellifolium* inferred from a nuclear gene. Mol Ecol 13: 1871–1882 15189210 10.1111/j.1365-294X.2004.02191.x

[jipb12383-bib-0005] Caicedo AL , Williamson SH , Hernandez RD , Boyko A , Fledel‐Alon A , York TL , Polato NR , Olsen KM , Nielsen R , McCouch SR ( 2007) Genome‐wide patterns of nucleotide polymorphism in domesticated rice. PLoS Genet 3: e163 10.1371/journal.pgen.0030163 PMC1994709 17907810

[jipb12383-bib-0006] Chou S ( 1948) China is the place of origin of rice. J Rice Soc China 7: 53–54

[jipb12383-bib-0007] Doi K , Izawa T , Fuse T , Yamanouchi U , Kubo T , Shimatani Z , Yano M , Yoshimura A ( 2004) *Ehd1*, a B‐type response regulator in rice, confers short‐day promotion of flowering and controls *FT*‐like gene expression independently of *Hd1* . Genes Dev 18: 926–936 15078816 10.1101/gad.1189604 PMC395851

[jipb12383-bib-0008] Eyre WA , Gaut RL , Hilton H , Feldman DL , Gaut BS ( 1998) Investigation of the bottleneck leading to the domestication of maize. Proc Natl Acad Sci USA 95: 4441–4446 9539756 10.1073/pnas.95.8.4441 PMC22508

[jipb12383-bib-0009] Fu D , Szűcs P , Yan L , Helguera M , Skinner JS , Zitzewitz JV , Hayes PM , Dubcovsky J ( 2005) Large deletions within the first intron in VRN‐1 are associated with spring growth habit in barley and wheat. Mol Genet Genomics 273: 54–65 15690172 10.1007/s00438-004-1095-4

[jipb12383-bib-0010] Fujino K , Sekiguchi H ( 2005) Mapping of QTLs conferring extremely early heading in rice (*Oryza sativa* L.). Theor Appl Genet 111: 393–398 15940510 10.1007/s00122-005-2035-3

[jipb12383-bib-0011] Fujino K , Wu J , Sekiguchi H , Ito T , Izawa T , Matsumoto T ( 2010) Multiple introgression events surrounding the *Hd1* flowering‐time gene in cultivated rice, *Oryza sativa* L. Mol Genet Genomics 284: 137–146 20607290 10.1007/s00438-010-0555-2

[jipb12383-bib-0012] Gao H , Zheng XM , Fei G , Chen J , Jin M , Ren Y , Wu W , Zhou K , Sheng P , Zhou F , Jiang L , Wang J , Zhang X , Wan JM ( 2013) *Ehd4* encodes a novel and Oryza‐genus‐specific regulator of photoperiodic flowering in rice. PLoS Genet 9: e1003281 23437005 10.1371/journal.pgen.1003281 PMC3578780

[jipb12383-bib-0013] Gao SQ , Chen M , Xia LQ , Xiu HJ , Xu ZS , Li LC , Zhao CP , Cheng XG , Ma YZ ( 2009) A cotton (Gossypium hirsutum) DRE‐binding transcription factor gene, *GhDREB*, confers enhanced tolerance to drought, high salt, and freezing stresses in transgenic wheat. Plant Cell Rep 28: 301–311 19005655 10.1007/s00299-008-0623-9

[jipb12383-bib-0014] Garris AJ , Tai TH , Coburn J , Kresovich S , McCouch S ( 2005) Genetic structure and diversity in *Oryza sativa* L. Genetics 169: 1631–1638 15654106 10.1534/genetics.104.035642 PMC1449546

[jipb12383-bib-0015] Ge S , Oliveira GC , Schaal BA , Gao LZ , Hong DY ( 1999) RAPD variation within and between natural populations of wild rice (*Oryza rufipogon*) from China and Brazil. Heredity 82: 638–644 10383685 10.1046/j.1365-2540.1999.00516.x

[jipb12383-bib-0016] Hottes AK , Freddolino PL , Khare A , Donnell ZN , Liu JC , Tavazoie S ( 2013) Bacterial adaptation through loss of function. PLoS Genet 9: e1003617 23874220 10.1371/journal.pgen.1003617 PMC3708842

[jipb12383-bib-0017] Izawa T ( 2007) Adaptation of flowering‐time by natural and artificial selection in *Arabidopsis* and rice. J Exp Bot 58: 3091–3097 17693414 10.1093/jxb/erm159

[jipb12383-bib-0018] Jakobsen IB , Easteal S ( 1996) A program for calculating and displaying compatibility matrices as an aid in determining reticulate evolution in molecular sequences. Comput Appl Biosci 12: 291–295 8902355 10.1093/bioinformatics/12.4.291

[jipb12383-bib-0019] Khush GS ( 1997) Origin, dispersal, cultivation and variation of rice. Mol Plant 35: 25–34 9291957

[jipb12383-bib-0020] Komiya R , Ikegami A , Tamaki S , Yokoi S , Shimamoto K ( 2008) *Hd3a* and *RFT1* are essential for flowering in rice. Development 135: 767–774 18223202 10.1242/dev.008631

[jipb12383-bib-0021] Koo BH , Yoo SC , Park JW , Kwon CT , Lee BD , An G , Zhang Z , Li J , Li Z , Paek NC ( 2013) Natural variation in *OsPRR37* regulates heading date and contributes to rice cultivation at a wide range of latitudes. Mol Plant 6: 1877–1888 23713079 10.1093/mp/sst088

[jipb12383-bib-0022] Le Corre Vr, Roux F, Reboud X ( 2002) DNA polymorphism at the *FRIGIDA* gene in *Arabidopsis thaliana*: Extensive nonsynonymous variation is consistent with local selection for flowering time. Mol Biol Evol 19: 1261–1271 12140238 10.1093/oxfordjournals.molbev.a004187

[jipb12383-bib-0023] Li X , Liu H , Wang M , Liu H , Tian X , Zhou W , Lv T , Wang Z , Chu C , Fang J , Bu Q. ( 2014) Combinations of *Hd2* and *Hd4* genes determine rice adaptability to Heilongjiang Province, northern limit of China. J Integr Plant Biol 57: 698–707 25557147 10.1111/jipb.12326

[jipb12383-bib-0024] Lu L , Yan W , Xue W , Shao D , Xing Y ( 2012) Evolution and association analysis of *Ghd7* in rice. PLoS ONE 7: e34021 22666315 10.1371/journal.pone.0034021 PMC3364234

[jipb12383-bib-0025] Posada D , Crandall KA ( 2001) Selecting the best‐fit model of nucleotide substitution. Syst Biol 50: 580–601 12116655

[jipb12383-bib-0026] Putterill J , Laurie R , Macknight R ( 2004) It's time to flower: The genetic control of flowering time. Bioessays 26: 363–373 15057934 10.1002/bies.20021

[jipb12383-bib-0027] Ross Ibarra J, Morrell PL, Gaut BS ( 2007) Plant domestication, a unique opportunity to identify the genetic basis of adaptation. Proc Natl Acad Sci USA 104: 8641–8648 17494757 10.1073/pnas.0700643104 PMC1876441

[jipb12383-bib-0028] Sabeti P , Schaffner S , Fry B , Lohmueller J , Varilly P , Shamovsky O , Palma A , Mikkelsen TS , Altshuler D , Lander ES ( 2006) Positive natural selection in the human lineage. Science 312: 1614–1620 16778047 10.1126/science.1124309

[jipb12383-bib-0029] Schaal B , Hayworth D , Olsen K , Rauscher J , Smith W ( 1998) Phylogeographic studies in plants: Problems and prospects. Mol Ecol 7: 465–474

[jipb12383-bib-0030] Second G ( 1982) Origin of the genic diversity of cultivated rice (Oryza spp.): Study of the polymorphism scored at 40 isozyme loci. Jpn J Genet 57: 25–57

[jipb12383-bib-0031] Slatkin M ( 1993) Isolation by distance in equilibrium and non‐equilibrium populations. Evolution 47: 264–279 10.1111/j.1558-5646.1993.tb01215.x 28568097

[jipb12383-bib-0032] Thompson PM , Toga AW ( 1997) Detection, visualization and animation of abnormal anatomic structure with a deformable probabilistic brain atlas based on random vector field transformations. Med Image Anal 1: 271–294 9873911 10.1016/s1361-8415(97)85002-5

[jipb12383-bib-0033] Tsuji H , Taoka Ki , Shimamoto K ( 2011) Regulation of flowering in rice: Two florigen genes, a complex gene network, and natural variation. Curr Opin Plant Biol 14: 45–52 20864385 10.1016/j.pbi.2010.08.016

[jipb12383-bib-0034] Wei X , Jiang L , Xu J , Zhang W , Lu G , Zhang Y , Wan J ( 2008) Genetic analyses of heading date of Japonica rice cultivars from Northeast China. Field Crops Res 107: 147–154

[jipb12383-bib-0035] Wei X , Qiao W , Yuan N , Chen Y , Wang R , Cao L , Zhang W , Yang Q , Zeng H ( 2013) Domestication and association analysis of *Hd1* in Chinese mini‐core collections of rice. Genet Resour Crop Evol 61: 121–142

[jipb12383-bib-0036] Wu W , Zheng XM , Lu G , Zhong Z , Gao H , Chen L , Wu C , Wang H , Wang Q , Zhou K , Wang JL , Wu F , Zhang X , Guo X , Cheng Z , Lei C , Lin Q , Jiang L , Wang H , Ge S , Wan J ( 2013) Association of functional nucleotide polymorphisms at *DTH2* with the northward expansion of rice cultivation in Asia. Proc Natl Acad Sci USA 110: 2775–2780 23388640 10.1073/pnas.1213962110 PMC3581972

[jipb12383-bib-0037] Xia X , Xie Z ( 2001) DAMBE: Software package for data analysis in molecular biology and evolution. J Hered 92: 371–373 11535656 10.1093/jhered/92.4.371

[jipb12383-bib-0038] Xue W , Xing Y , Weng X , Zhao Y , Tang W , Wang L , Zhou H , Yu S, Xu C, Li X, Zhang QF ( 2008) Natural variation in *Ghd7* is an important regulator of heading date and yield potential in rice. Nat Genet 40: 761–767 18454147 10.1038/ng.143

[jipb12383-bib-0039] Yano M , Katayose Y , Ashikari M , Yamanouchi U , Monna L , Fuse T , Baba T , Yamamoto K , Umehara Y , Nagamura Y , Sasaki T ( 2000) *Hd1*, a major photoperiod sensitivity quantitative trait locus in rice, is closely related to the Arabidopsis flowering time gene *CONSTANS* . Plant Cell 12: 2473–2483 11148291 10.1105/tpc.12.12.2473 PMC102231

[jipb12383-bib-0040] Zheng XM , Ge S ( 2010) Ecological divergence in the presence of gene flow in two closely related Oryza species (*Oryza rufipogon* and *O. nivara*). Mol Ecol 19: 2439–2454 20653085 10.1111/j.1365-294x.2010.04674.x

[jipb12383-bib-0041] Zhu Q , Zheng X , Luo J , Gaut BS , Ge S ( 2007) Multilocus analysis of nucleotide variation of *Oryza sativa* and its wild relatives: Severe bottleneck during domestication of rice. Mol Biol Evol 24: 875–888 17218640 10.1093/molbev/msm005

