# Supplementary figures and images for: Nonfunctional alleles of long‐day suppressor genes independently regulate flowering time
Source: J Integr Plant Biol. 2015 Sep 17;58(6):540–8. doi: 10.1111/jipb.12383 (PMC5049618; doi:10.1111/jipb.12383)

Fig.S1 Schematic diagrams of four nuclear loci and locations of the regions sequenced.

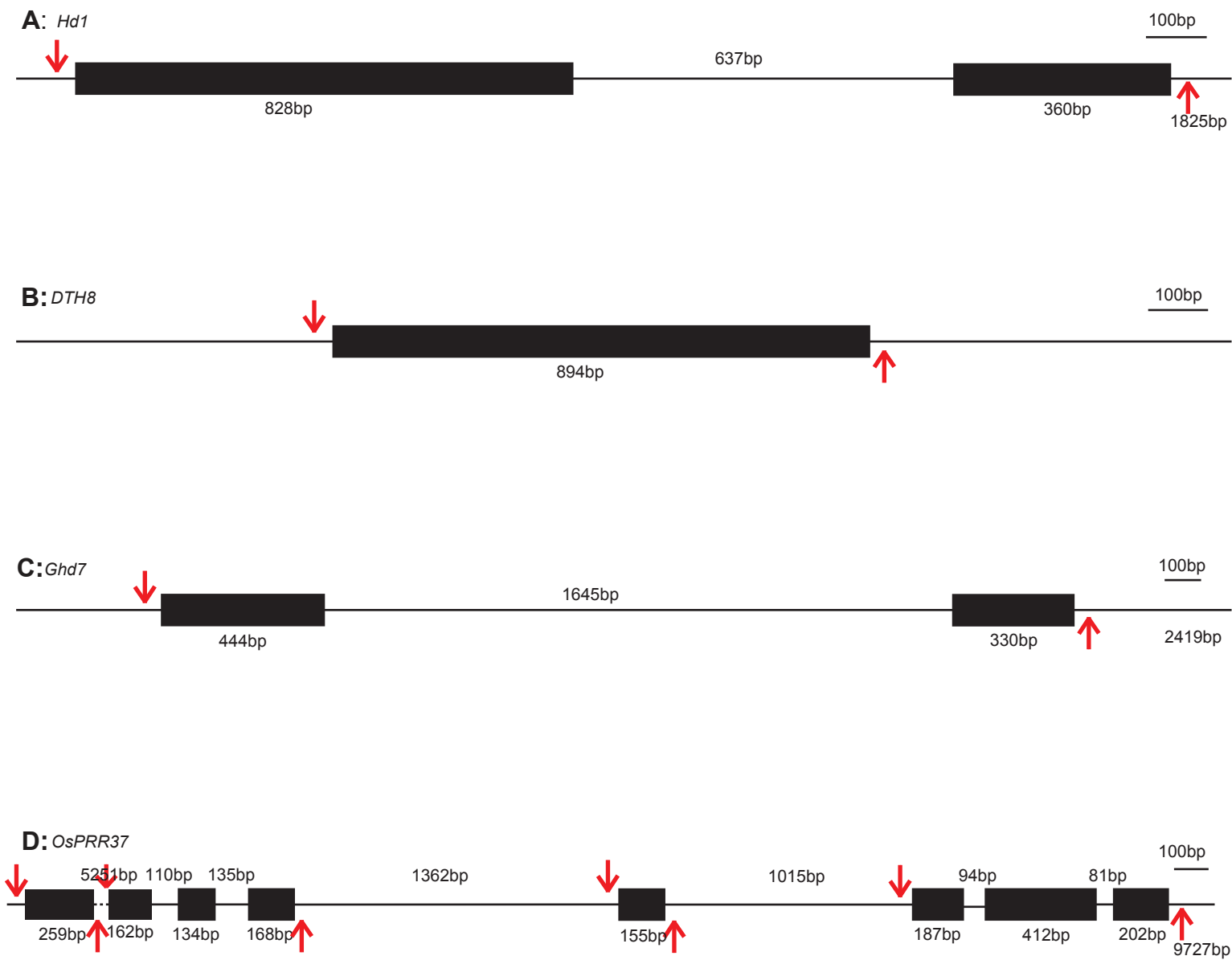

Supplement: Supplementary file 1 — Figure S1. Schematic diagrams of four nuclear loci and locations of the regions sequenced Exons are shown as black boxes; thin lines between black boxes refer to introns. Locations of primers for each fragment are sketched above the diagrams using red arrows. [file JIPB-58-540-s001.pdf]

Fig.S2 Distribution map of rice landraces carrying functional alleles .

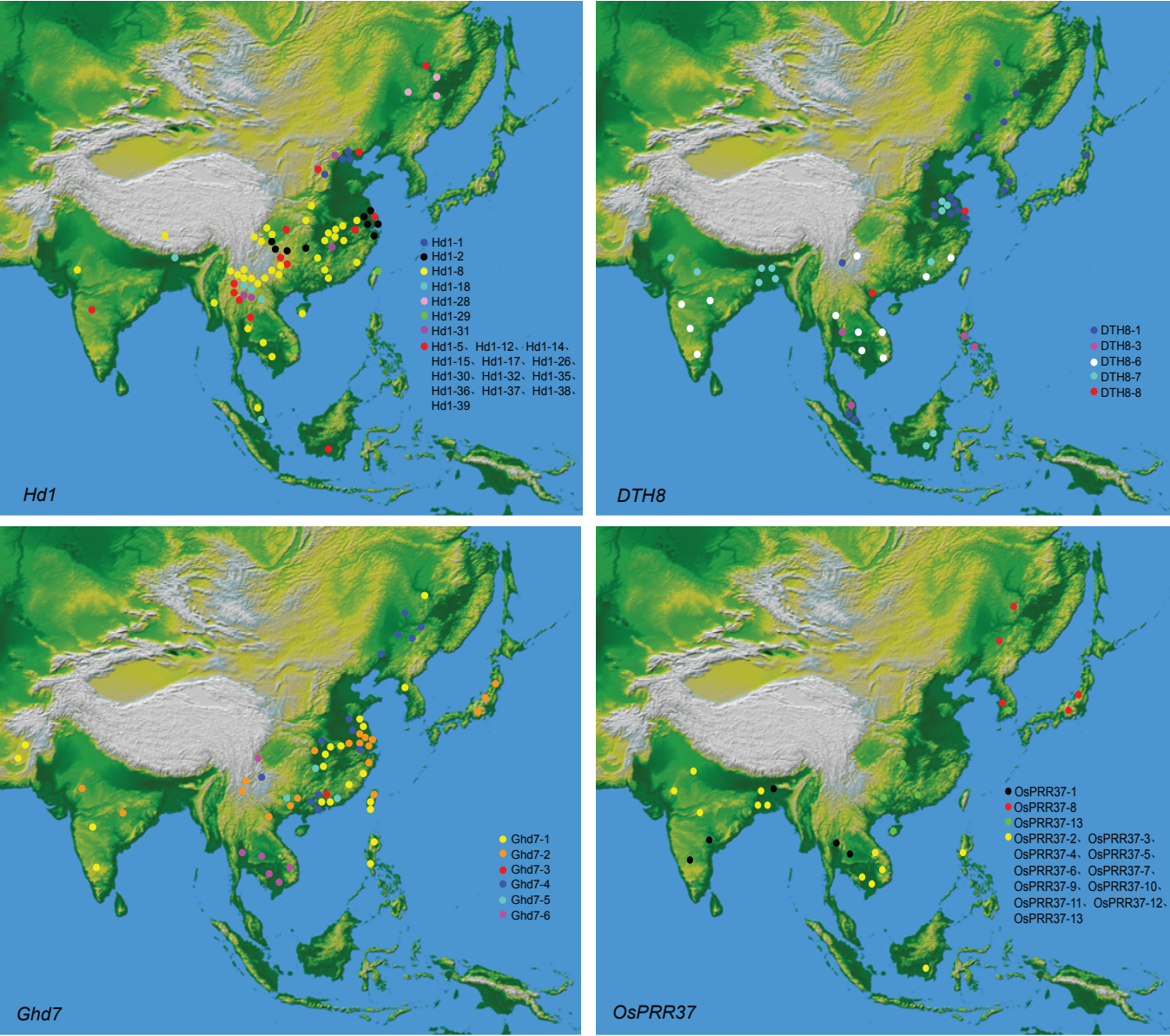

Supplement: Supplementary file 2 — Figure S2. Distribution map of rice landraces carrying the functional alleles (A) Hd1, (B) DTH8, (C) Ghd7 and (D) OsPRR37. Each circle represents one accession. Each color represents different haplotypes except that alleles with less than three varieties are shown in pink. [file JIPB-58-540-s002.pdf]
