# Supplementary material for: Nonfunctional alleles of long‐day suppressor genes independently regulate flowering time
Source: J Integr Plant Biol. 2015 Sep 17;58(6):540–8. doi: 10.1111/jipb.12383 (PMC5049618; doi:10.1111/jipb.12383)
Supplement: Supplementary file 3 — Table S1. Summary of nucleotide polymorphism of four loci [file JIPB-58-540-s003.docx]

Table S1 Summary of nucleotide polymorphism of four loci.

|  | *Hd1* | | *DTH8* | | *Ghd7* | | *OsPRR37* | |
| --- | --- | --- | --- | --- | --- | --- | --- | --- |
|  | Indica | Japonica | Indica | Japonica | Indica | Japonica | Indica | Japonica |
| *Hd* | 0.862 | 0.709 | 0.373 | 0.455 | 0.440 | 0.830 | 0.755 | 0.542 |
| *π* | 0.00522 | 0.00485 | 0.00395 | 0.00379 | 0.00257 | 0.00459 | 0.00239 | 0.00201 |
| *θ* | 0.00408 | 0.00331 | 0.00461 | 0.00297 | 0.00218 | 0.00283 | 0.00321 | 0.00272 |

*Hd*, Haplotype diversity; *π*, average number of pairwise nucleotide differences per site calculated on the silent sites; *θ*, Watterson’s estimator of per basepair calculated on the silent sites.
