# Supplementary material for: Nonfunctional alleles of long‐day suppressor genes independently regulate flowering time
Source: J Integr Plant Biol. 2015 Sep 17;58(6):540–8. doi: 10.1111/jipb.12383 (PMC5049618; doi:10.1111/jipb.12383)
Supplement: Supplementary file 4 — Table S2. The differences between the functional and nonfunctional alleles by one‐way ANOVA [file JIPB-58-540-s004.doc]

Table S2 The differences between the functional and nonfunctional alleles by one-way ANOVA

| Gene name | dfb | dfw | P-value |
| --- | --- | --- | --- |
| *Hd1* | 1 | 52 | 4.39*10^-6^ |
| *DTH8* | 1 | 66 | 0.027 |
| *Ghd7* | 1 | 69 | 5.69*10^-5^ |
| *OsPRR37* | 1 | 54 | 2.74*10^-5^ |

dfb: Degree of freedom between groups.

dfw: Degree of freedom within groups.
