# Supplementary material for: Nonfunctional alleles of long‐day suppressor genes independently regulate flowering time
Source: J Integr Plant Biol. 2015 Sep 17;58(6):540–8. doi: 10.1111/jipb.12383 (PMC5049618; doi:10.1111/jipb.12383)
Supplement: Supplementary file 6 — Table S4. The primer sequences used in this study [file JIPB-58-540-s006.doc]

Table S4 The primer sequences used in this study.

| Locus | Primer Sequence (5’-3’) |
| --- | --- |
| *Hd1* | F: atcgctggattcgacttgac;  R: tgtgattgttggccaaggta |
| *Ghd7* | F: ccccctacctttccctcat;  R: cgcactgtaattatctatctgaacc |
| *DTH8* | F: gatcgggtagggacgagaat;  R: atatgttttccccaatgtgc |
| *OsPRR37-1* | F: ggttagctgctgctgttggt;  R: ccataatctgcacccctttt |
| *OsPRR37-2* | F: gggaagttctttccccttgg;  R: cagtgacatggtgaactcttcc |
| *OsPRR37-3* | F: tcccctaacactgtgggaag;  R: cggtgataccccagaacaacc |
| *OsPRR37-4* | F: gttcagtgacatggccgata;  R: cgagggtgttgtacgaggtt |
